# Isolated Cutis Marmorata Telangiectatica Congenita in a Full-Term Neonate: A Case Report

**DOI:** 10.7759/cureus.82137

**Published:** 2025-04-12

**Authors:** Afnan Maashi, Ghadah Khormi, Laila Alali, Abdulaziz Rajhi, Najlaa Alsudairy

**Affiliations:** 1 General Practice, Jazan University, Jazan, SAU; 2 Dermatology, Prince Sultan Military Medical City, Jazan, SAU; 3 Family Medicine, National Guard Hospital, Jeddah, SAU

**Keywords:** case report, cmtc, congenital vascular anomaly, cutis marmorata telangiectatica congenita, differential diagnosis, livedo, neonate, skin discoloration

## Abstract

Cutis marmorata telangiectatica congenita (CMTC) is a rare condition present at birth, marked by a persistent, net-like purplish discoloration of the skin caused by abnormal blood vessels. We report a case of a full-term female neonate born following an uneventful pregnancy, who exhibited widespread, nonblanching, marble-like skin changes involving the trunk, limbs, and face. These findings were present at rest, did not resolve with warming, and were more prominent during crying. Physical examination revealed no associated limb asymmetry, neurologic abnormalities, or dysmorphic features. A comprehensive diagnostic workup, including laboratory tests, echocardiography, skeletal survey, and Doppler ultrasonography, ruled out neonatal lupus, Klippel-Trénaunay syndrome, and capillary malformation-arteriovenous malformation syndrome. Based on clinical features and exclusion of other conditions, a diagnosis of isolated CMTC was made. The patient was managed conservatively with parental education and scheduled follow-up. At six months of age, the skin changes had faded partially, and no complications or developmental delays were observed. This case emphasizes the importance of recognizing CMTC as a benign condition that warrants thorough evaluation to exclude associated anomalies and benefit from early, structured monitoring.

## Introduction

Cutis marmorata telangiectatica congenita (CMTC) is a rare congenital vascular anomaly characterized by persistent, reticulated, mottled skin discoloration that resembles physiological cutis marmorata but does not resolve with warming and is typically present at birth [1]. First described by Van Lohuizen in 1922, CMTC is considered a benign vascular malformation affecting the capillaries and venules of the dermis. Its incidence is unknown due to underreporting and misdiagnosis, but it is estimated to be rare, with fewer than 300 cases reported in the literature [1,2].

The hallmark of CMTC is a fixed, violaceous, and marbled skin pattern that may be segmental or widespread, often accompanied by telangiectasia and, in some cases, atrophy or ulceration. While many cases are isolated and self-limiting, CMTC may also be associated with extracutaneous abnormalities, including limb asymmetry, glaucoma, and developmental delay, necessitating a thorough evaluation and long-term follow-up [1-3]. The etiology of CMTC remains unclear, with sporadic occurrences being the most common. Genetic factors, including possible somatic mosaicism, have been proposed, although no specific gene mutation has been definitively linked to the condition [3,4].

## Case presentation

A full-term female neonate was born via spontaneous vaginal delivery to a 29-year-old gravida 2 para 1 mother at 39 weeks of gestation. The pregnancy was largely uneventful, with appropriate antenatal care and no reported exposure to teratogens, infections, or maternal illnesses. The mother had no history of diabetes, hypertension, or autoimmune disorders. The parents were nonconsanguineous, and there was no family history of vascular anomalies, dermatologic conditions, or congenital syndromes. Routine antenatal ultrasonography revealed no significant abnormalities. The Apgar scores at birth were 8 and 9 at one and five minutes, respectively, and birth weight was 3.2 kg (50th percentile for gestational age).

**Figure 1:**
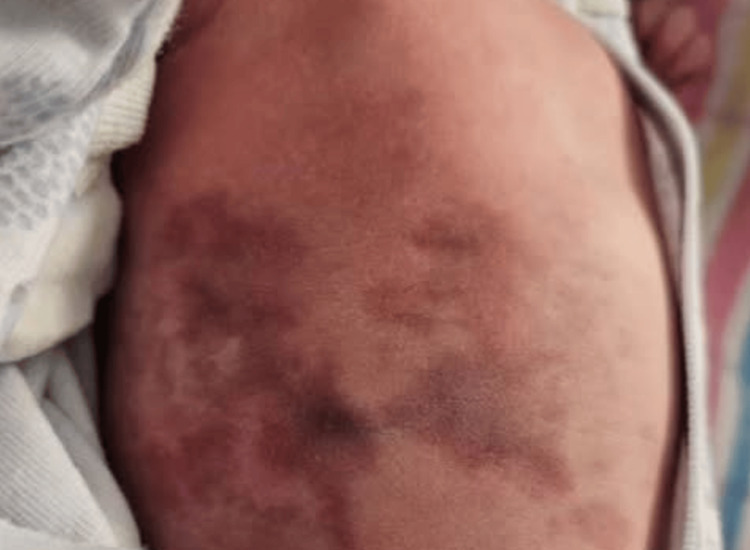
Clinical photograph of the neonate at birth showing widespread, reticulated, violaceous, and marble-like skin discoloration over the trunk, consistent with CMTC CMTC, cutis marmorata telangiectatica congenita

At birth, the neonate was noted to have a striking, persistent, reticulated, violaceous, and marble-like skin discoloration affecting the trunk (Figure 1). The skin findings were nonblanching and asymmetrical, with patchy erythematous and telangiectatic areas interspersed with livedoid reticulations. The skin changes were present at rest, did not resolve with warming, and were more prominent during episodes of crying. No ulceration, skin atrophy, or subcutaneous nodules were appreciated. There were no vesicular lesions, purpura, or bruising.

Physical examination was otherwise unremarkable. The neonate had a normal head circumference, normal tone, and spontaneous movements of all extremities. No limb length discrepancies, asymmetry, or joint deformities were observed. Cardiac, respiratory, and abdominal examinations were within normal limits. No dysmorphic facial features or neurological deficits were identified. Ophthalmologic and auditory screenings were unremarkable.

Given the distinctive cutaneous findings, initial differential diagnoses included CMTC, physiological cutis marmorata, neonatal lupus erythematosus, Klippel-Trénaunay syndrome (KTS), and capillary malformation-arteriovenous malformation (CM-AVM) syndrome. Laboratory investigations were performed to assess for systemic involvement and autoimmune etiologies. Complete blood count, basic metabolic panel, liver function tests, and inflammatory markers were all within normal ranges. Antinuclear antibodies and anti-Ro/SSA and anti-La/SSB antibodies were negative in both the neonate and the mother, reducing the likelihood of neonatal lupus.

A skeletal survey did not demonstrate any bony abnormalities or limb asymmetry. Echocardiography revealed a structurally normal heart with no evidence of septal defects, coarctation, or pulmonary hypertension. Cranial and abdominal ultrasound imaging did not identify any visceral anomalies. MRI of the brain was deferred due to the absence of neurologic symptoms. Doppler ultrasonography of the limbs and skin lesions showed no evidence of underlying venous or arterial malformations, thereby ruling out CM-AVM syndrome and vascular steal phenomena.

Based on the persistent reticulated vascular pattern, presence at birth, absence of systemic involvement, and exclusion of other vascular anomalies and autoimmune diseases, a diagnosis of CMTC was made. The diagnosis was clinical and supported by the characteristic appearance and distribution of the skin lesions.

Management was conservative, as the infant was otherwise healthy and feeding well. Parents were counseled extensively on the benign nature of CMTC in most cases and the importance of monitoring for potential complications, including ulceration, limb asymmetry, or delayed development. No pharmacologic treatment was initiated. Regular follow-up with dermatology and pediatrics was arranged.

**Figure 2:**
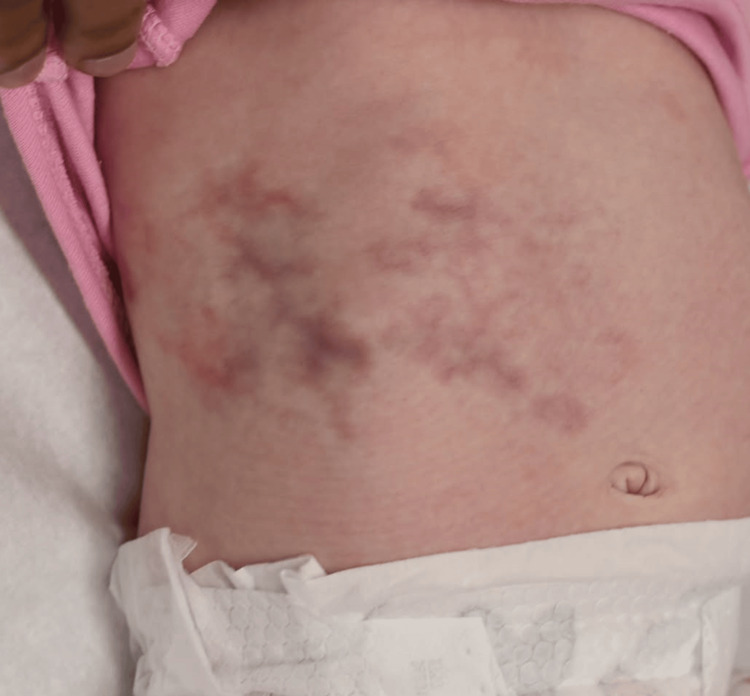
Follow-up clinical photograph at three months of age demonstrating partial fading of the cutaneous vascular pattern, with reduced intensity and distribution of the lesions

The infant was monitored weekly for the first month, then monthly for the next six months. Over the course of follow-up, the vascular skin changes remained largely unchanged but did not worsen. The infant continued to grow appropriately, met developmental milestones, and showed no signs of systemic involvement. At the three-month follow-up visit, the skin lesions persisted but began to fade slightly in intensity (Figure 2). There were no limb discrepancies, and neurologic examinations remained normal. The patient’s course has been uncomplicated to date, with continued follow-up planned to monitor for late-onset complications.

## Discussion

CMTC is a rare, sporadically occurring vascular anomaly of the skin with variable clinical presentation and prognosis. The case presented contributes to the limited body of literature on CMTC and underscores the importance of differentiating this benign condition from more serious systemic or syndromic vascular disorders [1-3]. In this neonate, a classical presentation of widespread reticulated erythema and telangiectasia, present at birth and nonresponsive to thermal changes, was critical for early clinical suspicion. Importantly, the absence of systemic features, limb asymmetry, and visceral involvement confirmed an isolated form of CMTC, which portends a more favorable outcome [4,5].

CMTC is thought to result from localized developmental abnormalities of the superficial cutaneous vasculature. While the precise etiology remains uncertain, some authors have hypothesized a pathogenic role for somatic mosaicism, potentially explaining the segmental distribution and sporadic occurrence. Unlike physiological cutis marmorata, which resolves with rewarming and is typically transient, CMTC lesions are persistent and may be associated with skin atrophy, ulceration, and, in some cases, underlying tissue hypoplasia. Diagnostic differentiation is essential, as CMTC may clinically resemble other conditions such as neonatal lupus erythematosus, KTS, CM-AVM syndrome, and livedo racemosa associated with systemic disease [1-4].

The differential diagnosis was systematically narrowed in this case through comprehensive clinical and imaging evaluation. The absence of maternal autoantibodies and lack of systemic involvement ruled out neonatal lupus. KTS was excluded by the absence of hypertrophy or venous malformations [4,5]. Doppler ultrasonography and echocardiography did not identify high-flow vascular lesions or arteriovenous malformations, decreasing the likelihood of CM-AVM syndrome. Notably, recent studies have identified mutations in RASA1 and EPHB4 in CM-AVM and Parkes Weber syndrome, respectively, highlighting the potential utility of genetic testing in complex cases. However, given the lack of systemic or syndromic features in our patient, genetic testing was not pursued.

The natural history of CMTC is generally benign, particularly in isolated cases. Several studies report partial or complete fading of skin lesions within the first two years of life. However, around 20-30% of patients may experience complications such as skin ulceration, limb length discrepancy, or neurologic delay, emphasizing the importance of early diagnosis and routine surveillance. In the current case, the absence of associated anomalies and reassuring developmental progress at six-month follow-up suggest a favorable prognosis [2-6].

Management of CMTC is primarily conservative. In uncomplicated cases, no pharmacological treatment is necessary. Education and reassurance of the family are paramount, particularly regarding the benign nature of the condition and its expected course. In select cases where ulceration or limb asymmetry occurs, multidisciplinary involvement including dermatology, orthopedics, and physical therapy may be indicated [1,4]. Our case reinforces the importance of a structured follow-up plan to monitor for delayed-onset complications.

## Conclusions

CMTC is a rare but generally benign congenital vascular anomaly that should be recognized early to distinguish it from more serious systemic or syndromic conditions. Accurate diagnosis relies on careful clinical evaluation, exclusion of differential diagnoses, and vigilant monitoring for associated complications. This case underscores the importance of a multidisciplinary, guideline-informed approach in managing neonates with congenital vascular lesions. The take-home message is that while CMTC often resolves spontaneously and requires no intervention in isolated cases, early recognition and structured follow-up are essential to ensure favorable outcomes and to promptly address any emerging complications.

## References

[1] Manna S (2024). A rare case of vascular malformation in India: cutis marmorata telangiectatica congenita. Cureus.

[2] Shah S, Santos da Cruz NF, Lopez-Font F, Kiryakoza L, Berrocal A (2024). Optical coherence tomography angiography in pediatric ocular cutis marmorata telangiectatica congenita: a case series. Am J Ophthalmol Case Rep.

[3] Belgemen-Ozer T (2024). Persistent cutis marmorata telangiectatica congenita associated with isolated hemihypertrophy and edema attacks. Clin Pediatr (Phila).

[4] Hokazono K, Urzedo AB, Dias PB, Dias NA (2024). Retinal abnormalities in a patient with cutis marmorata telangiectatica congenita. BMJ Case Rep.

[5] Ghotbabadi SH, Barzegar H, Abdolvand M (2024). Generalized cutis marmorata telangiectatica congenita or neonatal lupus? A case report and literature review. Clin Case Rep.

[6] Portal Buenaga M, Naharro Fernández C, Gómez Dermit V, de Las Cuevas Terán MI (2024). Newborn with cutis marmorata telangiectatica congenita. An Pediatr (Engl Ed).

